# Association of Metabolic Syndrome With Prevalence of Obstructive Sleep Apnea and Remission After Sleeve Gastrectomy

**DOI:** 10.3389/fphys.2021.650260

**Published:** 2021-03-31

**Authors:** Yufei Chen, Lijia Chen, Lingxia Ye, Jiabin Jin, Yingkai Sun, Ling Zhang, Shaoqian Zhao, Yifei Zhang, Weiqing Wang, Weiqiong Gu, Jie Hong

**Affiliations:** ^1^Department of Endocrine and Metabolic Diseases, Ruijin Hospital Affiliated to Shanghai Jiao Tong University School of Medicine, Shanghai, China; ^2^Shanghai Institute of Endocrine and Metabolic Diseases, Shanghai, China; ^3^Department of Endocrinology, The Second Affiliated Hospital of Zhejiang University School of Medicine, Hangzhou, China; ^4^Department of Pancreatic Surgery, Ruijin Hospital Affiliated to Shanghai Jiao Tong University School of Medicine, Shanghai, China

**Keywords:** metabolically healthy obesity, metabolically unhealthy obesity, metabolic syndrome, obstructive sleep apnea, laparoscopic sleeve gastrectomy

## Abstract

Obesity is an important risk factor for metabolic syndrome and obstructive sleep apnea (OSA). Bariatric surgery has been shown to effectively reduce weight and obesity-related comorbidities. However, the prevalence and severity of OSA in obese patients with different baseline metabolic states and the improvements of OSA after bariatric surgery remain unknown. The main aims of this study were to ascertain the prevalence of OSA in young Chinese obese patients with different metabolic states and to evaluate their respective OSA remission after laparoscopic sleeve gastrectomy. We first performed a cross-sectional study involving 123 metabolically healthy obese patients and 200 metabolically unhealthy obese patients (who had the same age and BMI ranges) to estimate the prevalence of OSA at baseline. Then we performed a retrospective study, which was registered at ClinicalTrials.gov (ref. NCT02653430) of 67 patients who underwent laparoscopic sleeve gastrectomy to evaluate the remission of OSA. Metabolically healthy and unhealthy obese patients had similar apnea-hypopnea index levels (16.6 ± 22.0 vs. 16.7 ± 18.7 events/h, *P* = 0.512) and prevalence of OSA (66.7% vs. 69.0%, *P* = 0.662). Male sex, age, waist circumference and lower liver-to-spleen ratio were independent risk factors for OSA. After laparoscopic sleeve gastrectomy, no difference was found in the decrease in body mass index (BMI) change (10.8 ± 4.8 vs. 10.8 ± 3.0 kg/m^2^, *P* = 0.996) or the decrease in the apnea-hypopnea index (18.9 ± 24.6 vs. 17.0 ± 24.0 events/h, *P* = 0.800). The remission of moderate-to-severe OSA was observed in the MHO (36.3%; 54.5–18.2%, *P* = 0.125) and MUO (32.2%; 66.1–33.9%, *P* = 0.001) patients. These results suggest that, in patients with obesity, metabolic syndrome does not add extra risk for the prevalence or severity of OSA. Both metabolically healthy and unhealthy obese patients could benefit equally from laparoscopic sleeve gastrectomy in terms of weight loss and obstructive sleep apnea remission.

## Introduction

The prevalence of obesity has continued to rise in prevalence at a rapid rate over the past decades. As of 2015, 2.2 billion people were overweight or obese worldwide, accounting for about one-third of the world’s total population (Reilly, 2017). It is well recognized that there is an obese phenotype that does not involve the typical metabolic disorders associated with obesity. This unique subset of obese individuals without metabolic syndromes (MetS) has been described as “benign obesity” or “metabolically healthy obesity (MHO).” Previous reports of this special obesity phenotype mostly focused on the risk of all-cause mortality, cardiovascular events, and diabetes, and the conclusions remain controversial. Some studies have suggested that obese individuals have increased risk of adverse outcomes even in the absence of metabolic abnormalities, especially over the long-term (Kramer et al., 2013), while other studies showed that MHO individuals had a lower risk of cardiovascular disease than their unhealthy counterparts (Meigs et al., 2006; Ogorodnikova et al., 2012; Phillips, 2013). As for the impact of MetS on other obesity-related comorbidities, there remains a lack of evidence.

Obstructive sleep apnea (OSA) is a sleep-related breathing disorder that is characterized by repeated episodes of partial or complete obstruction of the upper airway during sleeping. In the general adult population, the prevalence of OSA ranges from 9 to 38% (Senaratna et al., 2016), while in individuals with morbid obesity, it can affect >70% of the group and the prevalence increases with increasing body mass index (BMI) (Lopez et al., 2008). Epidemiological studies have indicated that OSA might also be an independent risk factor for stroke, ischemic heart disease, cardiac arrhythmia, and heart failure (Redline et al., 2010; Gottlieb et al., 2011) via several mechanisms, including oxidative stress, systemic inflammation, intrathoracic pressure changes, sympathetic activation and endothelial dysfunction (Malcolm and Stradling, 2010). Although some studies have indicated that there are higher rates of OSA among individuals with obesity or MetS (Drager et al., 2010; Leong et al., 2013), there are no previous studies comparing the prevalence of OSA in individuals with MHO and those with metabolically unhealthy obesity (MUO).

Bariatric surgery has been shown to be more effective at treating morbid obesity and its comorbidities than medicine and lifestyle changes (Madsbad et al., 2014; Schauer et al., 2017). Limited studies have demonstrated the beneficial effects of bariatric surgery regarding MetS remission (Ibrahim et al., 2017; Guilbert et al., 2018) and OSA remission (Peromaa-Haavisto et al., 2016; Timmerman et al., 2019). However, there are no previous studies comparing the impact of sleeve gastrectomy on weight loss and OSA remission between patients with or without MetS.

The aims of this study were to determine whether the presence of MetS increases the prevalence of OSA in young Chinese obese individuals and also to analyze whether the improvements in weight and OSA after laparoscopic sleeve gastrectomy are related to the preoperative metabolic state.

## Materials and Methods

### Study Population

The initial cross-sectional study included 323 obese patients (aged 14–40 years, BMI ≥ 30 kg/m^2^) who had been hospitalized at the Ruijin Hospital, Shanghai Jiao Tong University School of Medicine, from September 2011 to July 2019. Individuals with secondary causes of obesity were excluded. All participants were Han Chinese, had not taken antihypertensive, antidiabetic, or lipid-lowering drugs within the 3 months before study enrollment, and had provided informed consent.

Next, a retrospective study was conducted on 67 patients with obesity who had undergone laparoscopic sleeve gastrectomy from August 2013 to May 2019. These patients all met the criteria set out in “Comprehensive clinical practice guidelines for medical care of patients with obesity” published by the American Association of Clinical Endocrinologists and the American College of Endocrinology in 2016 (Garvey et al., 2016). We excluded subjects with serious systemic disease, alcohol or drug addiction, mental illness, and those who with a relatively high surgical risk. Surgeries were performed by the same group, with standardization of the processes and technique. Each patient provided written informed consent for their data to be used in this study. The study was registered at ClinicalTrials.gov (NCT02653430).

### Definition of MetS

Based on the National Cholesterol Education Program Adult Treatment Panel III (NCEP ATP III), the diagnosis of MetS was made when patients had ≥ 3 of the following components, which include a combination of categorical and borderline components: (1) abdominal obesity defined as waist circumference >102 cm in men or >88 cm in women, (2) triglycerides ≥1.7 mmol/L, (3) high-density lipoprotein cholesterol (HDL-C) <1.04 mmol/L in men or <1.30 mmol/L in women, (4) systolic blood pressure ≥130 mmHg and/or diastolic blood pressure ≥85 mmHg, and (5) fasting plasma glucose ≥5.6 mmol/L. Based on these criteria, 123 patients with obesity were classified into the MHO group while 200 patients were classified into the MUO group. Before laparoscopic sleeve gastrectomy, 11 patients were classified into the MHO group while 56 patients were classified into the MUO group.

### Diagnosis of OSA

All patients underwent standard overnight polysomnography (PSG) using the Alice-4 or Alice-5 Diagnostic Sleep System (Philips Healthcare/Respironics, Murrysville, Pennsylvania, United States). Apnea-hypopnea index (AHI) ≥5 events/h is defined as OSA according to the Clinical Practice Guideline for Diagnostic Testing for Adult Obstructive Sleep Apnea updated in 2016 (Kapur et al., 2017). Regarding the severity, AHI ≥ 5, ≥15, and ≥30 events/h was classified as mild, moderate, and severe, respectively. Postoperative PSG was performed for each patient who underwent sleeve gastrectomy to assess remission or persistence of OSA at a follow-up point between 6 and 12 months after surgery.

### Anthropometric and Biochemical Characteristics

A thorough medical history was recorded for each patient. Height, weight, waist, hip, and neck circumference and seated blood pressure were measured by an experienced physician. BMI was calculated as weight in kilograms divided by height in meters squared. Blood samples were taken in the morning (after an overnight fast for at least 10 h) for clinical biochemical analyses of lipid profiles and glycosylated hemoglobin (HbA1c). Additionally, an oral glucose tolerance test was performed (75 g of glucose was administered orally, and plasma glucose levels and serum insulin levels were detected at 0, 30, 60, 120, and 180 min). Homeostasis model assessment-estimated insulin resistance index (HOMA-IR) was calculated as fasting insulin (IU/ml) × fasting glucose (mmol/L)/22.5. Regarding the sleeve gastrectomy patients, data on the anthropometric and biochemical characteristics were collected again after bariatric surgery. Percentage of excess weight loss (%EWL) was calculated as:

$$\%\text{EWL}=\frac{\mathrm{preoperative}\mathrm{weight}-\mathrm{followup}\mathrm{weight}}{\mathrm{preoperative}\mathrm{weight}-\mathrm{ideal}\mathrm{weight}}\times 100$$

### Abdominal Computed Tomography (CT) Scan

Of the 323 patients in the initial study, 289 underwent general CT scans during a breath-hold at 5.0-mm collimation, 15.0-mm rotation-1 table speed (HQ mode, pitch 1:3), 120 kV(p), and Auto mA (Light speed QXi; GE Healthcare, Pittsburgh, Pennsylvania, United States). Visceral and subcutaneous adipose tissues were calculated using the single image at the L4–L5 vertebral interspace. Fat Scan software (N2 System) was used to determine the total, subcutaneous and visceral abdominal adipose tissue areas according to tissue density, and to calculate the visceral-to-total adipose tissue ratio. The liver-to-spleen ratio was determined by the mean value of the CT values measured in three regions of the liver and spleen, avoiding vessels, bile ducts, calcifications, and artifacts (Zeng et al., 2008).

### Statistical Analysis

Data analysis was performed with the statistical package SPSS (version 26.0; IBM, Armonk, New York, United States). Anthropometric, biochemical and PSG data are expressed as mean ± standard deviation or median (interquartile range) for normally or non-normally distributed variables. Categorical variables are expressed in terms of the frequency (and percentage) in each category and were compared using the chi-square test. Normally distributed continuous variables were compared using the independent-samples *t*-test, while non-normally distributed continuous variables were compared using the non-parametric test. To compare preoperative and postoperative data for each patient, the paired-samples *t*-test or Wilcoxon test was used for normally or non-normally distributed continuous variables. McNemar test was used to compare categorical variables before and after surgery. Binary logistic regression was used to identify independent predictors of the presence of OSA. A *P*-value of < 0.05 was considered significant.

## Results

### Clinical Characteristics and Metabolic Features in MHO and MUO Patients

According to the NCEP ATP III criteria, 323 patients with obesity were classified into the MUO group (*n* = 200) and MHO group (*n* = 123). Men represented 40.7% in the MHO group and 39.5% in the MUO group. The median age was 23 (20–27) years of MHO group and 25 (20–29) years of MUO group, respectively. Therefore, the age and sex distributions in the two groups were comparable. Smoking status was also similar between the two groups. There were no significant differences in body weight, BMI, neck, waist, and hip circumference, or waist-to-hip ratio. The mean BMI in the MHO and MUO groups was 38.0 ± 5.5 and 38.0 ± 4.8 kg/m^2^, respectively (*P* = 0.495). As expected, there were significant differences in blood pressure, fasting and 2-h glucose, fasting insulin and HbA1c. HOMA-IR and lipid levels were also higher in the MUO group except for low-density lipoprotein cholesterol (LDL-C) (Table 1). The MUO patients had a higher level of visceral abdominal adipose tissue (195.3 ± 127.0 vs. 160.6 ± 62.1 cm^2^, *P* = 0.002), a higher visceral-to-total adipose tissue ratio (28.4 ± 8.9% vs. 26.4 ± 7.2%, *P* = 0.031), and a lower liver-to-spleen ratio (0.74 ± 0.36 vs. 0.83 ± 0.32, *P* = 0.024).

**TABLE 1:** Clinical features and polysomnographic variables of obese patients with and without MetS.

Characteristic	MUO	MHO	*P*-value
Number	200	123	
Male sex n (%)	79 (39.5)	50 (40.7)	0.838
Age (years)	25 (20–29)	23 (20–27)	0.112
Smoker n (%)	21 (10.5)	8 (6.5)	0.223
Body weight (kg)	110.2 ± 19.00	108.8 ± 19.9	0.358
BMI (kg/m^2^)	38.0 ± 4.8	38.0 ± 5.5	0.495
Systolic blood pressure (mmHg)	137.1 ± 18.5	124.8 ± 15.5	< 0.001
Diastolic blood pressure (mmHg)	85.9 ± 12.3	78.2 ± 9.6	< 0.001
Fasting plasma glucose (mmol/L)	5.7 ± 1.4	5.4 ± 1.2	0.020
2-h plasma glucose (mmol/L)	9.1 ± 3.8	8.2 ± 3.1	0.014
Fasting serum insulin (μIU/ml)	26.9 ± 15.5	23.2 ± 12.9	0.015
2-h serum insulin (μIU/ml)	182.6 ± 139.7	180.7 ± 144.8	0.437
HOMA-IR	5.7 (4.2–8.1)	4.8 (3.0–6.7)	0.006
HbA1c (%)	5.7 (5.4–6.2)	5.6 (5.3–6.0)	0.039
Triglycerides (mmol/L)	2.0 (1.6–2.4)	1.2 (0.9–1.5)	< 0.001
Total cholesterol (mmol/L)	4.8 ± 1.1	4.6 ± 0.8	0.038
HDL-C (mmol/L)	1.0 ± 0.2	1.2 ± 0.2	< 0.001
LDL-C (mmol/L)	3.0 ± 0.8	2.9 ± 0.7	0.158
Total sleep time (min)	393 (340–448)	391 (350–439)	0.808
AHI (/h)	16.7 ± 18.7	16.6 ± 22.0	0.512
Lowest SaO_2_ (%)	84.0 (77.0–89.0)	85.0 (76.0–89.0)	0.557
Average SaO_2_ (%)	96.0 (94.3–97.0)	96.0 (95.0–97.0)	0.082
OSA	138 (69.0%)	82 (66.7%)	0.662
moderate to severe OSA	80 (40.0%)	42 (34.1%)	0.292

**Continuous variables are presented as mean ± standard deviation or median (interquartile range) and categorical variables are presented as N (%).**

### PSG Variables and Prevalence of OSA in MHO and MUO Patients

**FIGURE 1:**
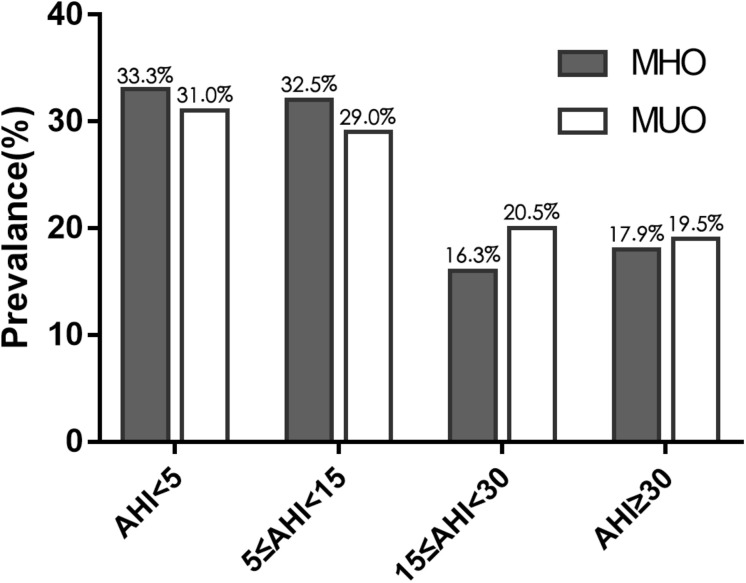
Prevalence of obstructive sleep apnea (OSA), of varying severity, in obese patients with and without MetS. Gray bars correspond to metabolically healthy obese patients and white bars correspond to metabolically unhealthy obese patients. The two groups had similar rates of the varying severities of OSA.

Regarding the PSG results of the 323 patients with obesity, no significant difference was found in total sleeping time. The MHO patients had a similar AHI to the MUO patients (16.6 ± 22.0 events/h vs. 16.7 ± 18.6 events/h, *P* = 0.512). The two groups also had similar minimum and mean oxygen saturation values. The prevalence of OSA was 66.7% (*n* = 82) in MHO and 69.0% (*n* = 138; *P* = 0.662) and the prevalence of moderate-to-severe OSA was 34.1% (*n* = 42) and 40.0% (*n* = 80; *P* = 0.292) in the MHO and MUO groups, respectively (Table 1). Moreover, the prevalence of each severity level of OSA did not differ between the two groups (Figure 1 and Supplementary Table 1).

In the stepwise binary logistic regression analysis, the presence of MetS was not an independent predictor of the presence of OSA (odds ratio 0.852, 95% confidence interval 0.487–1.491, *P* = 0.575), after adjusting for other variables (Table 2). In model 3, being male (odds ratio 2.491, 95% confidence interval 1.234–4.983, *P* = 0.010), age (odds ratio 1.075, 95% confidence interval 1.017–1.137, *P* = 0.010), waist circumference (odds ratio 1.051, 95% confidence interval 1.006–1.098, *P* = 0.025) and lower liver-to-spleen ratio (odds ratio 0.361, 95% confidence interval 0.156–0.835, *P* = 0.017) were the independent risk factors for the occurrence of OSA.

**TABLE 2:** The stepwise binary logistic regression model for predicting OSA.

Model 1			Model 2			Model 3		
Predictors	OR	*P*	Predictors	OR	*P*	Predictors	OR	*P*
Male sex	3.179	< 0.001***	Male sex	2.438	0.009**	Male sex	2.491	0.010*
Smoker	3.404	0.113	Smoker	3.519	0.105	Smoker	3.004	0.167
Age	1.100	< 0.001***	Age	1.088	0.001**	Age	1.075	0.010*
BMI	1.069	0.018*	BMI	1.032	0.559	BMI	1.029	0.610
MetS	0.975	0.926	MetS	0.993	0.980	MetS	0.852	0.575
			Neck circumference	0.977	0.554	Neck circumference	0.963	0.389
			Waist circumference	1.057	0.008**	Waist circumference	1.051	0.025*
			Hip circumference	0.971	0.244	Hip circumference	0.973	0.310
						Visceral-to-total abdominal adipose ratio	2.001	0.724
						Liver-to-spleen ratio	0.361	0.017*

*P < 0.05, **P < 0.01, ***P < 0.001.

### Changes in MetS Components and Other Clinical Features After Sleeve Gastrectomy

Regarding the follow-up analysis of the 67 sleeve gastrectomy patients, the mean postoperative follow-up time was 9.7 ± 2.8 months after surgery. The mean BMI decreased from 42.6 ± 7.7 to 31.9 ± 5.9 kg/m^2^ (*P* < 0.001). Significant improvements in MetS and each component were observed after surgery (Table 3). The components that improved most notably were fasting glucose and triglycerides levels, with percentage changes from 58.2 to 10.4% (*P* < 0.001) and 53.7 to 3.0% (*P* < 0.001), respectively. Waist circumference, blood pressure and HDL-C level also improved significantly. The preoperative prevalence of MetS was 83.6%, and there was rapid remission after surgery with a postoperative prevalence of 31.3% (*P* < 0.001).

**TABLE 3:** Baseline and postoperative clinical features and evolution of MetS.

Characteristic	Baseline	Follow-up	*P-*value
Body weight (kg)	119.6 ± 24.1	89.6 ± 19.2	< 0.001
BMI (kg/m^2^)	42.6 ± 7.7	31.9 ± 5.9	< 0.001
Waist circumference (cm)	123.6 ± 15.3	102.5 ± 14.4	< 0.001
Systolic blood pressure (mmHg)	141.0 ± 24.3	126.8 ± 20.3	< 0.001
Diastolic blood pressure (mmHg)	87.8 ± 15.7	76.5 ± 16.1	< 0.001
Fasting plasma glucose (mmol/L)	6.5 ± 2.0	4.8 ± 0.5	< 0.001
2-h plasma glucose (mmol/L)	10.9 ± 4.7	5.5 ± 2.1	< 0.001
HbA1c (%)	6.1 (5.5–7.0)	5.2 (4.9–5.6)	< 0.001
HOMA-IR	6.9 (4.1–10.9)	1.7 (1.2–2.6)	< 0.001
Triglycerides (mmol/L)	1.8 (1.4–2.1)	0.9 (0.8–1.2)	< 0.001
Total cholesterol (mmol/L)	4.8 ± 0.9	4.5 ± 0.9	0.001
HDL-C (mmol/L)	1.0 (0.9–1.2)	1.2 (1.0–1.4)	< 0.001
LDL-C(mmol/L)	3.0 ± 0.8	2.9 ± 0.8	0.099
AHI (/h)	31.9 ± 28.6	13.3 ± 15.8	< 0.001
Lowest SaO_2_ (%)	79.0 (63.0–83.0)	87.0 (82.0–90.0)	< 0.001
Moderate to severe OSA	43 (64.2%)	21 (31.3%)	< 0.001
MetS	56 (83.6%)	21(31.3%)	< 0.001
Abdominal obesity	67 (100.0%)	56 (83.6%)	0.001
High triglycerides level	36 (53.7%)	2 (3.0%)	< 0.001
Low HDL-C level	51 (76.1%)	36 (53.7%)	< 0.001
High blood pressure	44 (65.7%)	29 (43.3%)	0.004
High fasting glucose level	39 (58.2%)	7 (10.4%)	< 0.001
Number of components	3.9	1.7	< 0.001

**Continuous variables are presented as mean ± standard deviation or median (interquartile range) and categorical variables are presented as N (%).**

Additionally, both MHO and MUO patients achieved significant weight loss after surgery and there were no significant between-group differences in change in body weight or BMI (−10.8 ± 4.8 vs. −10.8 ± 3.0 kg/m^2^, *P* = 0.996). No statistically significant difference was observed in mean %EWL (63.2 ± 24.5 vs. 76.2 ± 28.9%, *P* = 0.124), indicating that surgery had a similar benefit of weight loss in the two groups (Table 4). In terms of surgical safety, 3 patients had severe postoperative complications included gastrointestinal obstruction and fistula. Gallstone disorders were the most common long-term adverse event, occurring in 14 patients.

**TABLE 4:** Postoperative evolution of clinical features and polysomnographic variables of obese patients with and without MetS.

	MUO (*n* = 56)			MHO (*n* = 11)			
Characteristic	Baseline	Follow-up	*P*-value	Baseline	Follow-up	*P*-value	*P*-value
Body weight (kg)	120.9 ± 23.9	90.9 ± 18.4	< 0.001	113.0 ± 25.2	83.3 ± 22.8	< 0.001	
BMI (kg/m^2^)	43.0 ± 7.8	32.2 ± 5.7	< 0.001	40.7 ± 7.0	29.9 ± 6.7	< 0.001	
δBMI (kg/m^2^)	10.8 ± 4.8	10.8 ± 3.0					0.996
Excess weight (kg)	50.5 ± 21.0	20.5 ± 15.6	< 0.001	43.9 ± 21.8	14.2 ± 19.4	< 0.001	
%EWL	63.2 ± 24.5	76.2 ± 28.9					0.124
AHI (/h)	32.7 ± 29.2	13.8 ± 16.0	< 0.001	27.8 ± 26.0	10.8 ± 15.3	< 0.001	
Lowest SaO_2_ (%)	75.0 (60.0–89.0)	89.0 (79.0–93.0)	< 0.001	79.0 (63.3–83.0)	87.0 (82.3–89.8)	< 0.001	
NO OSA	10 (17.9%)	20 (35.7%)	0.006	3 (27.3%)	6 (54.5%)	0.453	
Moderate to severe OSA	37 (66.1%)	19 (33.9%)	< 0.001	6 (54.5%)	2 (18.2%)	0.125	
δAHI (/h)	18.9 ± 24.6	17.0 ± 24.0					0.800

*Continuous variables are presented as mean ± standard deviation or median (interquartile range) and categorical variables are presented as N (%).*

### Postoperative Remission of OSA in MHO and MUO Patients

After surgery, AHI was dramatically reduced in the MUO (32.7 ± 29.2 to 13.8 ± 16.0/h, *P* < 0.001) and MHO patients (27.9 ± 26.0 to 10.8 ± 15.3/h, *P* < 0.001) patients, and there was no between-group difference in the decrease in AHI in both groups (18.9 ± 24.6 vs. 17.0 ± 24.0 events/h, *P* = 0.800). The remission of moderate-to-severe OSA was observed in the MHO (36.3%; 54.5–18.2%, *P* = 0.125) and MUO (32.2%; 66.1–33.9%, *P* < 0.001) patients, though there was no statistically significant difference between preoperative and postoperative prevalence of OSA in MHO patients (potentially due to the limited sample size in the MHO group). The remission rates of OSA and moderate-to-severe OSA were 37.5% vs. 23.9% and 66.7% vs. 54.1% in the MHO and MUO group, respectively. These results indicated that laparoscopic sleeve gastrectomy can lead to similar improvement in OSA in MHO and MUO patients.

## Discussion

In our study, we analyzed the relationship between metabolic state and the prevalence of OSA in obese patients and their remission after laparoscopic sleeve gastrectomy. To focus on the effects of obesity and surgery themselves, we selected a population of young, non-treated obese patients. We found that the prevalence and severity of OSA did not differ between the obese individuals with or without MetS (who had the same age and BMI ranges). Central obesity may play a more critical role in OSA than general obesity and metabolic state. More importantly, preoperative metabolic state did not affect the weight loss or improvement in OSA achieved by sleeve gastrectomy. With a mean interval of 9.6 months between surgery and follow-up assessment, the mean BMI decreased by 10.8 ± 4.8 kg/m^2^ and 10.8 ± 3.0 kg/m^2^ in the MUO and MHO patients, respectively. AHI decreased by 18.9 ± 24.6 events/h and 17.0 ± 24.0 events/h, respectively.

Although substantial evidence supported the strong association between increased BMI and metabolic disorders, recent studies have described the concept of the so-called “metabolically healthy obese,” who are a subpopulation of obese individuals with a normal metabolic phenotypes observed in a group of obese population. Several studies have supported the phenotypical absence of MetS in people with MHO (Bonora et al., 1998; Wildman et al., 2008). However, several obesity-related comorbidities, such as OSA, have not been systematically evaluated in people with MHO, leaving the question whether early intervention is valuable for MHO patients.

OSA is a common comorbidity of obesity. In the general adult population, the prevalence of OSA ranges from 9 to 38% (Senaratna et al., 2016), and obesity increases this number to as high as 70% (Lopez et al., 2008). Furthermore, the presence of other obesity-related metabolic disorders was independently associated with higher AHI (Heinzer et al., 2015), strongly suggesting an important role of OSA in the development of obesity-induced metabolic abnormalities. It is well known that the impaired upper airway anatomy is the primary cause of OSA. Although obesity is a major cause of a narrow pharyngeal airway as it can cause deposition of adipose tissue around the airway, obvious anatomical obstructions were not identified in many patients with obesity (Andrew et al., 2004). In the absence of an abnormal upper airway, the hypothesized mechanisms of obesity contributing to OSA vary widely. Upper airway inflammation seems to be one of the causes of OSA. Studies have shown inflammatory cell accumulation and increased cytokine levels in the upper airways (Marin et al., 2005; Vicente et al., 2016). Also, systemic inflammation augmented by obesity may cause pharyngeal myopathy and influence central control of the upper airway muscles (Hatipoğlu and Rubinstein, 2003). The similar degree of low-grade inflammation in the two groups may partly explain the comparable prevalence of OSA in MHO and MUO patients in our study. Second, many studies have revealed that central obesity is a major predisposing factor for OSA, as increased fat storage in upper airway structures can cause airway collapse (Patil et al., 2004; Francis et al., 2013; Harada et al., 2014). Consistently with these studies, our results also showed important effect of central obesity on the prevalence of OSA as weight circumference and liver/spleen ratio were independent risk factors for OSA. As men tend to gain weight more centrally than do women (Whittle et al., 1999), this may also partly explain why male sex is an important predisposing factor for OSA. These results suggest that the prevalence and severity of OSA in obesity are not influenced by metabolic state, and previous results indicate that OSA is more strongly correlated with central obesity than general obesity or metabolic state. Both MHO and MUO patients had a similar risk of OSA and its related metabolic abnormalities. These results highlight the importance of early screening for OSA and weight management among MHO individuals, to avoid the occurrence of related complications.

Bariatric surgery is the most effective treatment for obesity and also greatly improves the associated comorbidities. Bariatric surgery can lead to rapid and durable weight loss, which is often difficult to achieve with medications, diet, or physical exercises alone. Few studies have analyzed the relationship between bariatric surgery and remission of MetS. In a study performed by Martini et al. (2015), 75.8% of patients achieved remission, while 24.2% of patients showed persistence of MetS 1 year after Roux-en-Y gastric bypass. Guilbert et al. (2018) observed that the prevalence of MetS decreased from 100 to 6.34% 1 year after Roux-en-Y gastric bypass, and this decrease was still maintained at 2 years. Researchers also reported OSA remission in 70–80% patients in short and long-term after bariatric surgery (Casella et al., 2015; Timmerman et al., 2019). However, there are no studies focusing on the difference in weight loss and improvement of OSA in patients with or without MetS before surgery. We found that, after surgery, the prevalence of MetS decreased from 83.6 to 31.3% and all five components of MetS were improved, especially regarding the elevated triglycerides and fasting plasma glucose levels. Notably, sleeve gastrectomy led to a similar degree of weight loss in MHO and MUO patients. Excess weight was significantly decreased after surgery, with no difference was found in postoperative %EWL between the two groups. Additionally, the postoperative PSG assessments showed that AHI decreased from 32.7 to 13.8 events/h (*P* < 0.001) in MUO patients and from 27.8 to 10.8 events/h (*P* < 0.001) in MHO patients. There was no significant difference in the change in AHI (18.9 ± 24.6 vs. 17.0 ± 24.0 events/h, *P* = 0.800) and remission of moderate to severe OSA was observed in 32.2 and 36.3% of MUO and MHO patients, respectively. These results indicated that patients with obesity can achieve similar weight loss benefits and OSA remission after sleeve gastrectomy regardless of preoperative metabolic state after sleeve gastrectomy.

Recently, growing evidence has challenged the proposed existence of “healthy obese individuals,” as several studies found that MHO subjects were at increased risk of cardiovascular disease and type 2 diabetes compared to metabolically healthy normal weight individuals (Guy-Marino et al., 2015; Song et al., 2019). Our recent study also showed that the prevalence of polycystic ovarian syndrome was not influenced by the presence of MetS (Liang et al., 2017). All these results suggest that metabolically healthy obesity is not a benign condition, and that many complications have already developed before apparent changes in the metabolic state. Clinicians should take more initiative when treating MHO patients to avoid their transition to the MUO phenotype. This study also showed that whether or not patients presented with MetS before surgery, they could equally benefit from sleeve gastrectomy both regarding weight loss and OSA remission. Therefore, early bariatric surgery among obese patients with an apparently normal metabolic profile could be beneficial regarding improving OSA and avoiding conversion to the metabolically unhealthy phenotype.

A limitation of our hospital-based study is the small sample size and retrospective design of the follow-up study. Larger prospective studies in the future may provide us more powerful evidence regarding weight loss and remission rates of obesity-related comorbidities among MHO patients.

## Conclusion

In conclusion, our study illustrated that having a normal metabolic state did not decrease the prevalence or severity of OSA in patients with obesity. Weight management is equally important for both obese phenotypes. Both MHO and MUO patients who underwent laparoscopic sleeve gastrectomy experienced rapid and remarkable improvement in weight and OSA, suggesting that early bariatric surgery could be an higher-priority option when treating MHO patients.

## Data Availability Statement

The raw data supporting the conclusions of this article will be made available by the authors, without undue reservation.

## Ethics Statement

The studies involving human participants were reviewed and approved by Ethics committee of Ruijin Hospital Affiliated to Shanghai Jiao Tong University School of Medicine. Written informed consent to participate in this study was provided by the participants’ legal guardian/next of kin.

## Author Contributions

JH and WG contributed to study conception. YC and LC contributed to study design and drafted the manuscript. LZ, SZ, and LY contributed to analysis and interpretation of data. YZ, YS and WW contributed to critical revision. JJ contributed to the surgery. All authors contributed to acquisition of data.

## Conflict of Interest

The authors declare that the research was conducted in the absence of any commercial or financial relationships that could be construed as a potential conflict of interest.
